# Comparison of short-stretch bandage and long-stretch bandage for post-traumatic hand edema

**DOI:** 10.1016/j.ijscr.2023.108854

**Published:** 2023-09-20

**Authors:** Sheila Santandrea, Mariagrazia Benassi, Roberto Tedeschi

**Affiliations:** a Department of Biomedical and Neuromotor Sciences, Alma Mater Studiorum, University of Bologna, Bologna, Italy; b Department of Psychology Renzo Canestrani, University of Bologna, Viale Berti Pichat, 5, 40127 Bologna, Italy

**Keywords:** Hand edema, Orthopedic patients, Circular bandages, Finger flexion exercises, Treatment effectiveness

## Abstract

**Introduction and importance:**

Hand edema is a common post-surgical or traumatic complication in orthopedic patients, necessitating effective treatment interventions. This study aimed to investigate the effects of two different types of bandages, along with finger flexion exercises, on managing hand edema.

**Case presentation:**

Our orthopedic patients with post-surgical or traumatic hand edema and three non-edematous hands were enrolled in the study. A mixed model effect with fixed factors of time (pre-post) and bandage type (M, C, N), and random factors of hand, edema, fingers, and phalanges was applied. The bandage types were circular with short elastic bandage (M) and circular with elastic bandage (C). Finger flexion exercises involved alternating contractions of extrinsic and intrinsic flexors. Randomization ensured unbiased allocation to bandage types.

**Clinical discussion:**

The M bandage demonstrated a significant reduction in hand edema by effectively moving free fluids, reinforcing tissue hydrostatic pressure, and facilitating venous and lymphatic flow. On the other hand, the C bandage did not produce significant pre-post differences in hand circumference.

**Conclusions:**

The combination of a circular bandage with finger flexion exercises shows promise in reducing hand edema in orthopedic patients. Particularly, the stiff bandage M exhibited superior efficacy compared to the elastic one C in reducing hand circumference. These findings provide valuable insights for clinical practice, offering an effective strategy for managing hand edema and promoting better patient outcomes.

## Highlights

• Effective “M” short elastic bandage: “M” bandage significantly reduces edema.
• Enhanced patient care: Promising edema management strategies.
• Clinical impact is achievable: Improved hand edema care outcomes

## 1 Introduction

Post-traumatic edema is caused by an accumulation of fluid in the extracellular and intracellular spaces [1]. This accumulation of interstitial fluid is controlled by vascular and non-vascular processes that influence capillary filtration and lymphatic drainage [2]. Under normal conditions, microcirculatory homeostasis is maintained through a balance between capillary filtration and lymphatic drainage. Edema, which is the accumulation of fluid in the extracellular matrix, can occur due to excessive filtration or insufficient lymphatic system function. In traumatic edema, the lymphatic drainage system is typically functioning normally, and the accumulation of fluid in the interstitial space is secondary to vasodilation and hyperfiltration resulting from tissue damage [1]. There is an initial phase (acute edema or inflammatory phase) where the excess fluid (transudate) consists mainly of water and electrolytes. Therefore, the edema is fluid, soft, and easily mobilizable [1]. “Pitting edema” may be observed. In acute edema, if the lymphatic system is intact and not overwhelmed, it is capable of absorbing the excess fluid [3]. However, if the amount of interstitial fluid exceeds the transport capacity of the lymphatic system, temporary lymphatic overload occurs. In the proliferation phase, the consistency of the edema changes, and the interstitial fluid becomes enriched with proteins due to the deposition of fibrin and neocollagen. The presence of proteins in the extracellular matrix further promotes hyperfiltration. Moreover, the increased fluid expelled by the capillaries stimulates the production of additional fibrin by fibrocytes through mechanotransduction, creating a vicious cycle [3]. This represents the second phase (persistent edema or fibroplasia) in which the excess fluid is defined as exudate. The body tries to eliminate the excess proteins through macrophages, which attract fibroblasts, causing fibrosis in the area. The deposition of fibrin leads to adhesions between structures, resulting in a more viscous, non-pitting edema [2]. In the third phase (maturation phase), if edema is still present, it becomes hard and fibrotic, known as “brawny edema” [2]. Post-traumatic edema is part of the physiological tissue repair process, but it can become pathological when it persists beyond the inflammatory phase [4]. In particular, a swollen hand loses elasticity, strength, and precision in fine motor tasks, and the excess fluid can compress peripheral nerves (motor and sensory) [5]. Furthermore, persistent edema can lead to stiffness, flexion contractures, functional loss, and long-term disability [5]. Compression is one of the treatment modalities used for edema. In the acute phase, it is aimed at limiting the available space for fluid accumulation. In the fibroplasia phase, it slows down scar formation and fibrosis by reducing blood flow and creating local hypoxia. In the maturation phase, it helps soften fibrous connective tissue and maintain the reduction achieved during therapy [2]. Compression bandaging is characterized by various properties, including “pressure,” “layers,” “components,” and “elasticity” [6]. In particular, elasticity can be divided into two categories: short-stretch bandages (non-elastic or rigid) and long-stretch bandages (elastic). The first category has an elongation capacity ranging from 10 % to 100 %, while the second category has an elongation >100 % [6,7]. The first category has low resting pressure, making it well-tolerated, but high working pressure with elevated peaks during upright standing, walking [7], and exercises [8]. In contrast, the second category has a working pressure that is not much higher than the resting pressure [6], if not the same [9], and a continuously high resting pressure, making it less tolerable. In the literature, compression has been studied for various clinical conditions [10,11]. Specifically, numerous studies have compared the use of short-stretch bandages with long-stretch bandages for venous leg ulcers [12,13] and lymphedema (primary and secondary) [14]. Regarding post-traumatic edema, some studies have investigated the use of compression, albeit to a lesser extent compared to the aforementioned conditions, for the lower limb [15] and the upper limb (wrist-hand) [16]. However, there is no literature comparing bandages with different stiffness. Therefore, this case series aims to observe the effectiveness of a short-stretch bandage compared to a long-stretch bandage on post-traumatic orthopedic hand edema. The study enrolled adult orthopedic patients with post-traumatic edema of the fingers. The results of this study will contribute valuable insights into the management of post-traumatic hand edema in orthopedic patients and provide evidence-based guidance for selecting appropriate bandaging techniques.

## 2 Case presentations

### 2.1 Participants (Table 1)

• The study included 4 orthopedic patients with hand edema of post-surgical or traumatic origin.
• There were 5 affected hands, with 1 patient having bilateral involvement, and 3 hands without edema.

**Table 1:** Patient characteristics and conditions.

Patient	Age	Hand	Diagnosis	Condition
Pz1	62	Right	Post-trapeziectomy and suspension arthroplasty	Edematous right hand, treated at 1 month post-surgery
Pz2	53	Right	Seronegative arthritis	Bilateral edema
Pz3	47	Right	Post-fracture F2	Evaluation at 5 weeks, edematous left hand
Pz4	66	Right	Post-radial head prosthesis with radial nerve palsy	Treated at 1 month post-surgery, edematous left hand

### 2.2 Intervention

• The treatment protocol involved two different types of bandages: a. Circular bandage with short elastic bandage (Bendaggio circolare con benda a corta elasticità) - denoted as “M” b. Circular bandage with elastic bandage (Bendaggio circolare con benda elastica) - denoted as “C”

### 2.3 Exercise

• Participants performed finger flexion exercises, involving alternating contractions of extrinsic and intrinsic flexors.
• Each exercise consisted of 10 repetitions, holding each contraction for 5 s.
• Participants performed the exercise every 2 h during waking hours.

### 2.4 Randomization

Randomization was performed using a blocked design, ensuring that each hand had two fingers assigned to G1 (“M” bandage group) and two fingers assigned to G2 (“C” bandage group). This approach was employed to maintain equal representation of individual subject and pathology-related variables in both groups, as well as equal adherence to the treatment. Randomization was accomplished using allocation lists 13,325 and 25,330 generated through Randomization.com, ensuring an unbiased and robust allocation process.

### 2.5 Bandage composition

• The composition of the bandages used was as follows (fig):
  o “M” bandage: Composed of 42 % cotton, 29 % polyamide, and 29 % viscose.
  o “C” bandage: Composed of synthetic rubber fibers TNT, with an extensibility of >100 %.

### 2.6 Outcomes

The assessment of hand circumference was performed at P1, P2, and P3 of the four long fingers (2nd, 3rd, 4th, and 5th) of both hands, using a flexible tape measure with reduced height and equipped with a counterweight for standardizing tension

### 2.7 Study phases

**Fig. 1:**
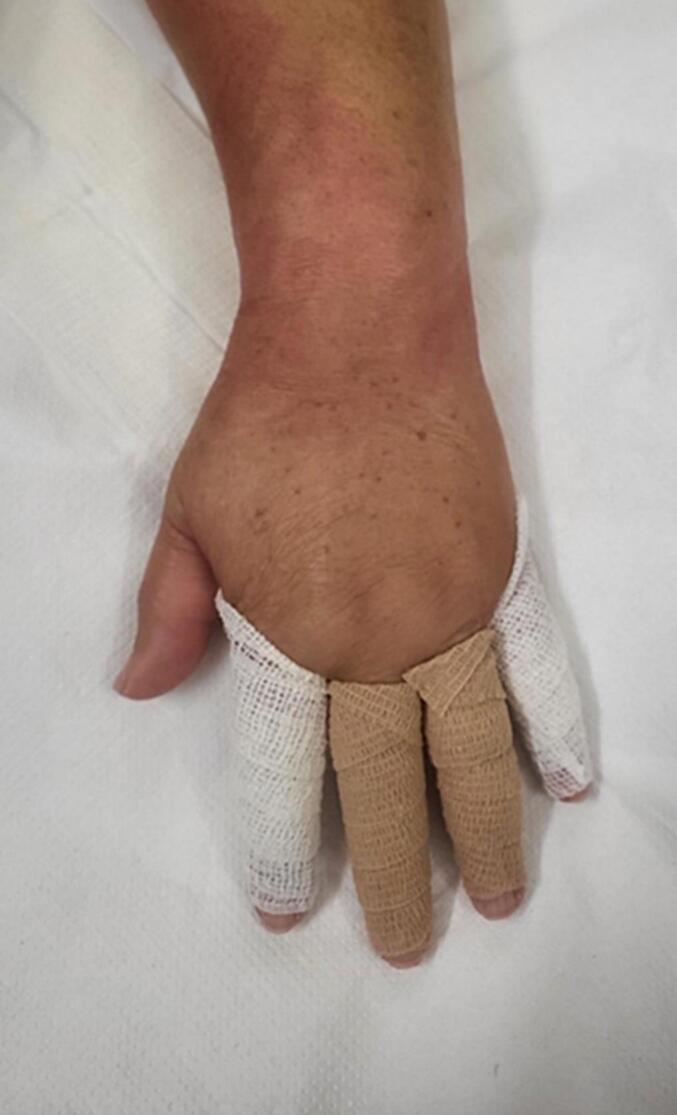
Different bandage.

Recruitment of participants included adult orthopedic/rheumatologic patients with hand edema who had no contraindications to active finger flexion and extension. Exclusion criteria involved individuals with lymphatic system dysfunctions. The assessment of hand circumference was performed at P1, P2, and P3 of both hands at T0. Participants's fingers were randomly allocated to two groups: G1 with “M” bandage and G2 with “C” bandage, and received training in finger flexion exercises. Re-evaluation occurred at T1 (24 h later). The methods described above were used to investigate the effects of different bandages (Fig. 1) and finger flexion exercises on post-surgical or traumatic hand edema. The study included edematous hands, with random allocation of the fingers to the two different bandage types. The fingers of the non-edematous hand were not bandaged, but the circumference assessment was evaluated as a control group N. The findings from this study could provide valuable insights into the treatment of hand edema in orthopedic patients. This case series has been reported in line with the PROCESS 2020 Guideline [17].

## 3 Clinical findings

Description: The table displays the mean values of hand edema circumference across different conditions. “No” indicates the absence of a bandage, and “None” represents the control group where no bandage was applied. Time is categorized as “0” for pre-treatment and “1” for post-treatment. Two types of bandages are compared: “C” signifies a circular bandage with an elastic bandage, while “M” indicates a circular bandage with a short elastic bandage. The results suggest that the M bandage was more effective in reducing edema compared to both the C bandage and the absence of a bandage, especially for cases with edema present at both time points.

**Fig. 2:**
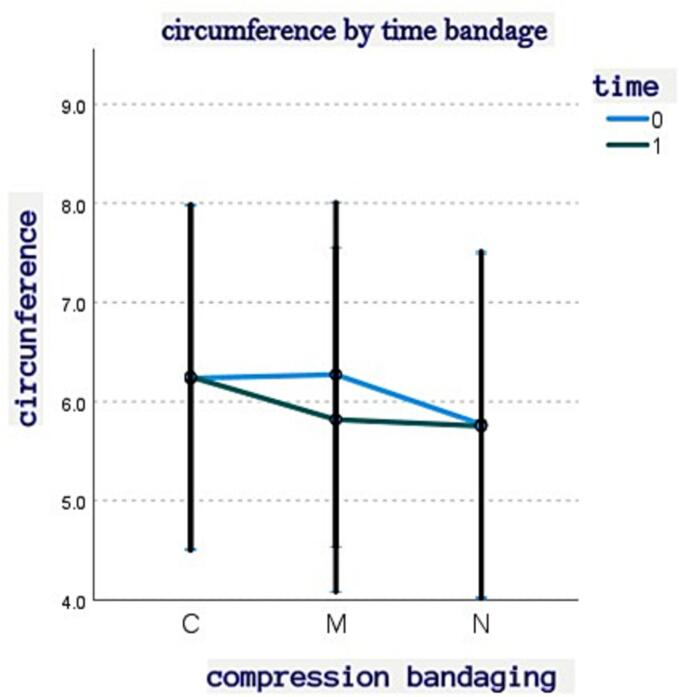
Circumference by time bandage.

A mixed model effect was applied with fixed factors of time (pre-post) and type of bandage (M, C, N), and random factors for hand, edema, fingers, and P (phalanx). From the analysis, an interaction effect between time, type of bandage, and the presence of edema emerged. The differences in circumference between the edema and non-edema conditions varied in the pre-post effect depending on the type of treatment. Bandage M showed a significant decrease in circumference compared to bandage C, which did not show significant pre-post differences. The results suggest that bandage M is particularly effective in reducing edema compared to the other types of bandages (C and N). This interaction effect is visually illustrated in the following (Fig. 2, Table 2). It is important to note that the random factors, including hand, edema, fingers, and phalanx, may contribute to overall data variability and should be considered in the comprehensive evaluation of the results.In conclusion, the findings suggest that bandage M may be the preferred choice for reducing hand edema compared to bandages C and N. However, it is essential to acknowledge the limited sample size, and further research with larger and more representative samples is required to confirm these results and further explore the effects of each treatment in managing hand edema.

**Table. 2:** Comparison of Hand Edema Circumference with Different Bandages and Time Points.

Edema	Time	Bandage	Mean	Std. error	95 % CI lower	95 % CI upper
No	0	N	5.770	0.883	4.028	7.512
	1	N	5.753	0.881	4.015	7.490
Yes	0	C	6.234	0.880	4.498	7.971
		M	6.271	0.880	4.535	8.007
	1	C	6.249	0.881	4.512	7.987
		M	5.817	0.880	4.081	7.554

## 4 Follow-up and outcomes

The findings of this study have several implications for both research and clinical practice in the management of hand edema.

1. Further Research: Given the study's small sample size and specific focus on post-surgical or traumatic hand edema, it is important to conduct further research with larger and more diverse participant samples. Future studies could explore the effectiveness of the examined interventions in different types of edema and various clinical conditions, thereby enhancing the generalizability of the findings. Additionally, there are already studies available that evaluate the different bandaging techniques on patients with lymphatic dysfunctions. The novelty of this study lies in investigating these techniques specifically in orthopedic patients, who often receive inadequate attention regarding edema management. Expanding research to include this population will contribute valuable insights to the field of hand rehabilitation and provide a more comprehensive understanding of edema treatment strategies.
2. Long-Term Effects: Although the study demonstrated positive outcomes in reducing hand edema during the 24-hour treatment period, it would be valuable to investigate the long-term effects of the interventions. Understanding the sustainability of the improvements over an extended period would provide clinicians with insights into the durability of the treatment effects. Specifically, exploring the timing for potential replacement of the rigid bandage, considering its role in maintaining pressure and managing edema, would be beneficial for optimizing treatment protocols. Further research with extended follow-up periods would be instrumental in elucidating the longevity of the treatment benefits and guiding clinical decision-making.
3. Comparative Studies: Conducting comparative studies between different bandaging techniques and exercise regimens could help identify the most effective interventions for managing hand edema. Comparing the circular bandage with short elastic bandage (M) and the circular bandage with elastic bandage (C) against other commonly used treatments could provide valuable information for clinical decision-making.
4. Mechanistic Investigations: Further research could delve into the underlying mechanisms of how the bandaging interventions and finger flexion exercises impact edema reduction. Understanding the specific physiological processes involved would enhance our knowledge of the treatment's effectiveness and may lead to the development of more targeted interventions.
5. Clinical Guidelines: Based on the study's positive outcomes, the results could potentially inform the development of clinical guidelines for the treatment of hand edema. Incorporating these interventions into standard care protocols could improve patient outcomes and overall quality of life.
6. Individualized Treatment Plans: As the study highlighted variability among participants, developing individualized treatment plans based on patient characteristics, such as the severity of edema and the type of edema, could optimize treatment effectiveness.
7. Rehabilitation Protocols: Integrating finger flexion exercises as part of rehabilitation protocols for post-surgical or traumatic hand edema patients may promote functional recovery and enhance overall treatment outcomes.

## 5 Discussion

This study delved into the effects of two distinct bandage types, alongside finger flexion exercises, in the context of post-surgical or traumatic hand edema within orthopedic patients. Employing a rigorous mixed model analysis, we factored in fixed variables encompassing time (pre-post) and bandage type (M, C, N), while accommodating random variables related to hand, edema, fingers, and phalanges. Our findings strongly suggest that the circular bandage, in conjunction with a short elastic bandage (referred to as “M”), may hold potential for reducing hand edema. This bandage exhibited several advantageous characteristics, including the facilitation of fluid drainage to targeted regions, the reinforcement of tissue hydrostatic pressure, improved venous and lymphatic flow, and the depolymerization of the water-collagen complex. Consequently, the application of the M bandage led to a statistically significant reduction in hand circumference. In stark contrast, the circular bandage with an elastic bandage (referred to as “C”) did not yield significant differences in hand circumference before and after treatment (as presented in Table 2). Moreover, our study unveiled the added benefits of incorporating finger flexion exercises in conjunction with the M bandage. These exercises, designed to enhance muscle contractions, improve blood flow, and bolster overall hand function, not only contributed to a significant reduction in edema but also maintained the gains achieved during the treatment period. However, it is crucial to highlight that the treatment duration in this study was 24 h. Future investigations should consider longer-term effects, as preliminary indications suggest that elastic bandages may offer advantages over rigid ones in comprehensively tracking edema dynamics over extended periods. In summary, our research underscores the superior effectiveness of the M bandage over the commonly employed C bandage in the context of hand edema management among orthopedic patients. These findings can provide valuable guidance to clinicians seeking informed strategies for addressing hand edema. Nevertheless, to solidify these outcomes and delve deeper into the long-term implications, further research involving larger and more diverse participant cohorts is imperative.

### 5.1 Strengths and limitations

#### 5.1.1 Strengths

1. Controlled Study Design: The study utilized a controlled design with randomization, which enhanced the internal validity of the findings. Random allocation of participants to different interventions reduced potential selection bias and ensured that the groups were comparable at baseline.
2. Interventions Combination: The study investigated the effects of two different types of bandages (circular bandage with short elastic bandage and circular bandage with elastic bandage) in conjunction with finger flexion exercises. This combination of interventions reflects a comprehensive and multifaceted approach to managing hand edema.
3. Clinically Relevant Outcome: The primary outcome measure, hand circumference, is a clinically relevant and objective indicator of edema reduction. The study's focus on functional outcomes is valuable in evaluating the effectiveness of the interventions in real-life scenarios.
4. Inclusion of Non-Edematous Hands: The inclusion of non-edematous hands for comparison provided a useful control group, enabling researchers to distinguish the effects of the interventions specifically on edematous hands.
5. Mechanistic Insights: The study explored the potential mechanisms behind the effectiveness of the interventions, such as moving free fluids, reinforcing hydrostatic pressure, and facilitating venous and lymphatic flow. This mechanistic investigation contributes to a better understanding of the treatment's underlying processes.

#### 5.1.2 Limitations

1. Small Sample Size: The study's limited sample size, consisting of only four orthopedic patients with hand edema, may limit the generalizability of the findings to a larger population. The small sample size reduces the statistical power of the study and increases the risk of Type II errors.
2. Specific Population: The study focused on post-surgical or traumatic hand edema, which restricts the applicability of the findings to other types of edema or non-traumatic conditions. Including a more diverse range of edema etiologies would enhance the study's external validity.
3. Lack of Follow-Up: The study's short duration and lack of long-term follow-up do not provide insight into the sustainability of the treatment effects. Longitudinal assessments would be valuable in understanding the persistence of improvements beyond the treatment period.
4. Limited Outcome Measures: While hand circumference is a relevant outcome measure, incorporating additional objective measures, such as edema volume or functional assessments, could provide a more comprehensive evaluation of treatment efficacy.
5. Single-Center Study: The study was conducted at a single center, which may limit the generalizability of the findings to other settings or healthcare facilities with potentially different patient populations and treatment protocols.
6. Missing Data: The study did not explicitly address missing data or the methods used to handle it, which could introduce bias and impact the study's results and conclusions.

## 6 Conclusions

The study suggests that the circular bandage with short elastic bandage (M) was more effective in reducing hand edema in orthopedic patients compared to the circular bandage with elastic bandage (C). Combining these interventions with finger flexion exercises showed promising results for treatment outcomes and functional recovery. However, further research with larger and more diverse participant samples is needed to validate these findings and explore the long-term effects of these interventions.

## Consent

Written informed consent was obtained from the patient for publication of this case report and accompanying images. A copy of the written consent is available for review by the Editor-in-Chief of this journal on request.

## Ethical approval

Ethical approval is not a requirement at our institution for reporting individual cases or case series.

## Funding

There is no source of funding.

## Author contribution

SS proposed the revision project and identified the framework. SS proposed the methodology. SS identified the research strategy. RT extracted and analysed the data. RT and MGB supervised the methodology. All authors conducted the revision and developed the first and subsequent drafts of the manuscript.

## Guarantor

Roberto Tedeschi.

## Research registration number

Not applicable.

## Declaration of competing interest

Authors state no conflict of interest.
